# miRNA regulation of cytotoxic effects in mouse Sertoli cells exposed to nonylphenol

**DOI:** 10.1186/1477-7827-9-126

**Published:** 2011-09-14

**Authors:** Jin-Sung Choi, Jung-Hwa Oh, Han-Jin Park, Mi-Sun Choi, Se-Myo Park, Seung-Jun Kang, Moon-Ju Oh, Seung Jun Kim, Seung Yong Hwang, Seokjoo Yoon

**Affiliations:** 1 Division of Research and Development, Korea Institute of Toxicology, 19 Shinsung-ro, Yuseong, Daejeon, 305-343, Korea; 2 GenoCheck Co., Ltd. & Hanyang University, Sa 3-dong, Sangnok-gu, Ansan, Gyeonggi-do, 426-791, Korea

## Abstract

**Background:**

It is known that some environmental chemicals affect the human endocrine system. The harmful effects of endocrine disrupting chemical (EDC) nonylphenol (NP) have been studied since the 1980s. It is known that NP adversely affects physiological functions by mimicking the natural hormone 17 beta-estradiol. In the present study, we analyzed the expression of miRNAs and their target genes in mouse Sertoli TM4 cells to better understand the regulatory roles of miRNAs on Sertoli cells after NP exposure.

**Methods:**

Mouse TM4 Sertoli cells were treated with NP for 3 or 24 h, and global gene and miRNA expression were analyzed using Agilent mouse whole genome and mouse miRNA v13 arrays.

**Results:**

We identified genes that were > 2-fold differentially expressed in NP-treated cells and control cells (*P *< 0.05) and analyzed their functions through Gene Ontology analysis. We also identified miRNAs that were differentially expressed in NP-treated and control cells. Of the 186 miRNAs the expression of which differed between NP-treated and control cells, 59 and 147 miRNAs exhibited 1.3-fold increased or decreased expression at 3 and 24 h, respectively. Network analysis of deregulated miRNAs suggested that *Ppara *may regulate the expression of certain miRNAs, including miR-378, miR-125a-3p miR-20a, miR-203, and miR-101a, after exposure to NP. Additionally, comprehensive analysis of predicted target genes for miRNAs showed that the expression of genes with roles in cell proliferation, the cell cycle, and cell death were regulated by miRNA in NP-treated TM4 cells. Levels of expression of the miRNAs miR-135a* and miR-199a-5p were validated by qRT-PCR. Finally, miR-135a* target gene analysis suggests that the generation of reactive oxygen species (ROS) following exposure to NP exposure may be mediated by miR-135a* through regulation of the Wnt/beta-catenin signaling pathway.

**Conclusions:**

Collectively, these data help to determine NP's actions on mouse TM4 Sertoli cells and increase our understanding of the molecular mechanisms underlying the adverse effects of xenoestrogens on the reproductive system.

## Background

Nonylphenol (NP) is a xenobiotic compound that is generated by the degradation of nonylphenol ethoxylates (NPEs). NPEs are used worldwide as oil-soluble detergents and emulsifiers (in the production of anionic detergents), lubricants, antistatic agents, high-performance textile-scouring agents, emulsifiers for agrochemicals, antioxidants (in the manufacture of rubber and plastics), and lubricant oil additives [1]. Due to their widespread use, significant quantities of incompletely degraded NPs reach sewage treatment works. Because of its high hydrophobicity, low solubility, and accumulation in the environment, NP is found in many parts of the world in rivers, water, soil, groundwater, sediment, the atmosphere, sewage sludge, and even drinking water. Because of its toxic effects, it has been banned in Canada and the EU and is being carefully monitored in many other countries [2].

NP is a known disruptor of the endocrine system. It acts by mimicking natural hormones, thereby inhibiting or stimulating the endocrine system [2]. Specifically, NP mimics the natural hormone 17β-estradiol and tends to compete for estrogen receptor binding sites [3,4]. 17β-estradiol influences the development and maintenance of male and female sex characteristics [5]. Recently, it was also found that NP has anti-androgenic activity and can disturb the proper function of androgens. Androgens are essential for normal development, including that of the reproductive systems in males [6]. In addition its effects on the endocrine system, NP also has immunoregulatory properties, and influences the cell cycle, apoptosis in neural stem cells, and the proliferation of breast cancer cells [1]. Like this, NP can induce the reproductive toxicity by disturbing the function of endogenous estrogens via receptor mechanism and also cause the cell death by modulating cellular mechanism via its phenolic group.

The results of several investigations suggest that NP can induce cell death by inhibiting the activity of endoplasmic reticulum Ca^2+ ^pump [7]; however, the molecular mechanisms behind NP's actions remain unclear. To investigate the toxic mechanisms of NP in male reproductive system, we previously performed gene expression profiling using testis tissues from mice that were repeatedly exposed to NP [8]. We found that genes with roles in spermatogenesis, such as *Odf1 *and *Sox *family genes, were differentially expressed in the testes following exposure to NP. It is thought that expression of these genes may be regulated by sophisticated mechanisms involving epigenomic regulators such as miRNAs.

MicroRNAs (miRNAs) are small non-coding regulatory RNAs, about 22 nucleotides in length. They contain 2-8-nucleotide sequences known as 'seed' regions that bind to completely or partially complementary sequences in the 3'-untranslated regions (3'-UTRs) of target mRNAs [9]. miRNAs regulate gene activity by repressing the translation of target mRNAs or triggering their degradation [10]. They are expressed in a wide range of tissues and during all stages of development in many species. To date, hundreds of miRNAs have been identified, each of which can regulate several genes. Computational predictions indicate that more than one third of all human genes may be miRNA targets [11]. miRNAs have been functionally linked to embryonic development, cell proliferation, differentiation, apoptosis and stress responses. Moreover, they also have been studied in the context of cancer and neurodegenerative diseases such as Alzheimer's disease and Parkinson's disease [12,13]. However, few reports have described the effects of toxicants on miRNA expression profiles, although recent investigations suggest the possible use of miRNAs as toxicity biomarkers [14,15].

The aim of the present study was to investigate the effects of NP on miRNA expression. In this study, we treated in the mouse TM4 Sertoli cells with 10 μg/mL NP. Sertoli cells play a pivotal role in the regulation of spermatogenesis [16,17] and TM4 is a well-established mouse cell line that retains Sertoli cell function. After exposing TM4 cells to NP, we monitored the expression of genes and miRNAs at early and late time point using microarrays. We performed comprehensive analyses of gene and miRNA expression to better understand the regulatory roles of miRNAs in the response to NP exposure.

## Methods

### Cell culture and NP treatment

Mouse TM4 Sertoli cells were obtained from the Korean Cell Line Bank (KCLB; Seoul, Korea). TM4 cells were grown at 37°C in a humidified 5% CO_2 _atmosphere in Dulbecco's Modified Eagle Medium (DMEM) containing 10% fetal bovine serum (FBS) and antibiotics (100 U/mL penicillin, 100 μg/mL streptomycin). DMEM, FBS, penicillin/streptomycin and trypsin-EDTA were obtained from HyClone (Logan, UT, USA). Cells were subcultured every 2-3 days. NP purchased from SUPELCO (Bellefonte, PA, USA) was suspended in DMSO (Sigma-Aldrich, St. Louis, MO, USA) and applied to TM4 cells at appropriate concentrations.

### Cell viability assay

Cell viability was determined by a formazan assay using the Cell Counting Kit-8 (CCK-8) (Dojindo Laboratory, Kumanoto, Japan). TM4 cells suspended in culture medium were seeded in 24-well culture plates at a density of 1 × 10^5 ^cells per well. They were then washed and exposed to vehicle (control) or varying concentrations of NP for 24 h. Following NP treatment, 100 μL of CCK-8 solution was added to each well and the cells were incubated at 37°C for a further 1 h. Absorbances at 450 nm were then measured using an ELISA reader (Bio-Rad, Japan). All experiments consisted of three independent replicates, each performed at least in triplicate. Data are presented as the mean ± SD.

### RNA isolation

Cells treated with 10 μg/mL NP for 3 or 24 h were harvested and total RNA extracted using the TRIzol reagent (Invitrogen, CA, USA) according to the manufacturer's instructions. RNA yield was quantified using a NanoDrop (NanoDrop, USA) and RNA quality using a 2100 Bioanalyzer (Agilent Technologies, CA, USA). Extracted RNA samples were stored at -80°C prior to microarray analysis. miRNA microarray experiments were conducted within 2 days of RNA extraction.

### miRNA expression microarray analysis

miRNA microarray analysis was performed using Agilent mouse miRNA v13 arrays. An miRNA Complete Labeling and Hyb Kit (Agilent Technologies) was used to label total RNA samples (100 ng) containing miRNAs with Cy3. Labeled samples were then applied to Agilent mouse miRNA v13 arrays and covered with an A4 hybridization mixer. The arrays were then incubated for 12 h at 42°C in a MAUI 12-bay Hybridization System. The hybridized slides were washed by the method described above (Gene expression microarray analysis) and dried through centrifugation (3,000 rpm, room temperature) [18].

### Gene expression microarray analysis

Agilent mouse genome 4 × 44 K arrays were use to profile gene expression in TM4 cells exposed to NP. Total RNA samples (30 μg) from cells treated with 10 μg/mL NP were reverse transcribed using SuperScript (Invitrogen, CA, USA) in conjunction with dCTP labeled with Cyanine 3 (Cy3) or Cyanine 5 (Cy5) (NEN Life Science, CA, USA). The two resulting cDNA samples were combined in equal amounts. Each cDNA mixture was then applied to an Agilent mouse genome 4 × 44 K array and covered with an A4 hybridization mixer (BioMicro Systems Inc., UT). The arrays were then incubated for 12 h at 42°C in a MAUI 12-bay Hybridization System (BioMicro Systems Inc) to allow hybridization. They were next washed in wash buffer 1 (2 × SSC, 0.1% SDS) for 5 min, in wash buffer 2 (1 × SSC) for 5 min, and lastly, in wash buffer 3 (0.2 × SSC) for 10 min. Finally, they were dried through centrifugation (3,000 rpm, room temperature) [19].

### Microarray data analysis

Hybridized arrays were scanned using a scanner (Agilent Technologies) and the resulting scanned images analyzed using the Feature Extraction Software (v10.7; Agilent Technologies). Expression profiling, clustering, gene ontology and GeneNetworks analyses were performed using GeneSpring GX (v11.0; Agilent Technologies). The microarray data were subjected to statistical analysis. Data acquisition and cut-off creation were used to obtain quantified data from the scanned images. For each spot, the median intensity rather than the mean, was calculated. Functional analysis of selected genes was performed through Gene Ontology (GO) analysis and using the KEGG Pathway Database, GeneSpring, and JAK software (GenoCheck Co. Ltd, Korea). Hierarchical clustering analysis of global gene expression patterns in different samples was performed using the GeneSpring Cluster 3.0 software (Euclidean distance and an average linkage algorithm were used). Data for chosen genes were analyzed by 1-way ANOVA analysis with time as the selected parameter. Multiple testing correction was achieved by Benjamini Hochberg false discovery rate (FDR).

### Prediction of miRNA targets and their functional classification

miRNA target genes were identified using the GeneSpring software and then analyzed using the Ingenuity Pathway Analysis (IPA) software to identify their biological function and canonical pathways. Fisher's exact test was used to calculate statistical significance. We then compared the results for miRNAs with those for gene expression.

### Quantitative RT-PCR

We selected two miRNAs that showed aberrant expression changes and validated and quantified them in a TaqMan miRNA assay (Applied Biosystems, CA, USA), performed using miRNA-specific primers according to the manufacturer's instructions. TM4 cells were treated with 10 μg/ml NP for 24 h and total RNA was isolated using the TRIzol reagent. RNA quality was assessed using a 2100 Bioanalyzer. Duplicate total RNA samples were prepared from control and NP-treated cells and analyzed in triplicate by real-time PCR analysis, performed using a 7900 HT Fast Real-Time PCR System (Applied Biosystems). miRNA levels were calculated relative to average levels of target miRNAs and compared to controls after normalization to an internal control, snoRNA202. The amount of snoRNA202 in each sample was calculated from a standard curve.

## Results and Discussion

### Cytotoxic effects of NP on TM4 cells

**Figure 1:**
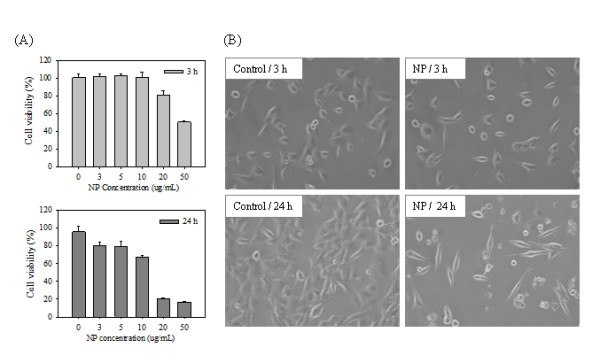
**Cell viability of TM4 cells treated with NP and phase contrast microscope images**. **(A) **Measurement of viability using CCK-8. All experiments consisted of three independent replicates, each performed at least in triplicate. Data are presented as the mean ± SD. **(B) **Morphological observation of NP-treated TM4 cells. TM4 cells were treated with 10 μg/ml NP.

We performed a cell viability assay using CCK-8 solution to evaluate the cytotoxic effects of NP on TM4 cells. The viability of TM4 cells was measured after growth in presence of NP (0, 3, 5, 10, 20 or 50 μg/mL) for 3 or 24 h. As shown in Figure 1-(A), the viability of TM4 cells exposed to NP for 24 h decreased, in a dose-dependent manner. Notably, we found that cytotoxicity was markedly increased in cells treated with NP at a concentration of 20 or 50 μg/mL at 24 h at both time points. We chose to use NP at a concentration of 10 μg/mL (which reduced cell viability to approximately 70% at 24 h) in subsequent microarray analyses. The cytotoxic effects of NP were also confirmed by phase contrast microscopic analysis of TM4 cells treated with 10 μg/mL NP for 3 and 24 h. At 3 h, there was no significant changes of cell morphology but cells exposed to NP for 24 h displayed morphological changes (e.g., irregular shape and cytoplasmic blebbing), were sparse, and detached from the culture plates on which they were growing (Figure 1-(B)). In addition, LDH assay showed that LDH released into the culture medium was significantly elevated after NP treatment (data not shown). The cytotoxic effect of NP in Sertoli cells was also consistent with the result of previous investigations [20].

### miRNA expression profiling in NP-treated TM4 cells

**Figure 2:**
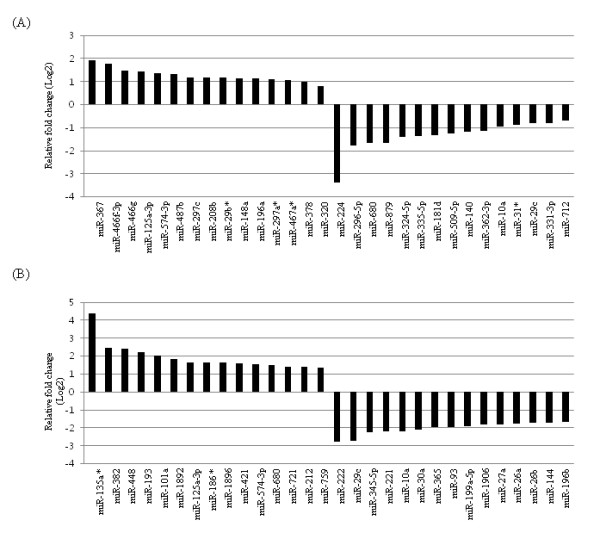
**Expression fold changes for the most highly deregulated miRNAs in NP-treated TM4 cells**. (A) 3 h. (B) 24 h.

To explore miRNA regulation in Sertoli cells following NP treatment, we performed miRNA expression analysis using Agilent mouse miRNA v13 arrays. miRNA expression profiling showed that exposure to NP significantly altered miRNA expression levels at 3 h and 24 h. In total, 186 miRNAs were > 1.3-fold up- or down-regulated following exposure to NP (59 at 3 h and 147 at 24 h). We chose the cut-off condition with 1.3-fold (*P *< 0.05) because a cut-off fold-change of 1.3 was considered as reasonable magnitude and statistical *P *value is more important rather than the intensity for the analysis of miRNA expression changes. In addition, the false positive miRNAs were filtered out in the present study using multiple test corrections as described the method section. Hierarchical clustering of differentially expressed miRNAs revealed well-coordinated expression between sample groups (Additional file 1, Figure S1). The miRNAs that displayed differential expression in NP-treated cells at each time point are listed in Table 1. Most miRNAs the expression of which was altered in NP-treated cells at 24 h were down-regulated. Of the 186 miRNAs the expression of which was altered, nine were up-regulated at both time points (miR-125a-3p, miR-297c, miR-421, miR-452, miR-483, miR-574-3p, miR-574-5p, miR-669a, miR-720) and 11 were down-regulated at both time points (let-7g, miR-107, miR-10a, miR-15a, miR-15b, miR-199b*, miR-26a, miR-29c, miR-324-5p, miR-331-3p, miR-342-3p). Two of the down-regulated miRNAs, miR-15a and miR-15b, regulate the cell cycle by controlling the expression of BCL2 and several cyclin family members (D1, D2, E1) [21,22]. miR-125a-3p and miR-107 are also involved in cell cycle and it is known that their expression is regulated by PPARA [23]. The functions of the other miRNAs the expression of which was altered at both time points remain unclear. Figure 2 shows the miRNAs the expression of which differed the most between NP-treated and control cells. Changes in miRNA expression at 3 h ranged from 11-fold down-regulation (miR-224) to 3.8-fold up-regulation (miR-367), and at 24 h from 7-fold down-regulation (miR-222) to 20.6-fold up-regulation (miR-135a*; Figure 2).

**Table 1:** miRNAs the expression of which was altered following exposure to NP for 3 or 24 h

	miRNAs	
Time point	Up-regulated	Down-regulated
3 h (n = 59)	(n = 35)	(n = 24)
	miR-125a-3p, miR-145, miR-148a, miR-181c, miR-196a, miR-202-5p, miR-208b, miR-23b, miR-297a*, miR-297c, miR-29b*, miR-30c, miR-320, miR-367, miR-378, miR-383, miR-421, miR-450b-5p, miR-451, miR-452, miR-466f-3p, miR-466g, miR-467a*, miR-467e*, miR-470*, miR-471, miR-483, miR-484, miR-487b, miR-574-3p, miR-574-5p, miR-669a, miR-697, miR-720, miR-764-5p	let-7g, miR-107, miR-10a, miR-140, miR-15a, miR-15b, miR-181d, miR-224, miR-199b*, , miR-26a, miR-296-5p, miR-29c, miR-31*, miR-324-5p, miR-331-3p, miR-335-5p, miR-342-3p, miR-362-3p, miR-466a-3p, miR-509-5p, miR-680, miR-689, miR-712, miR-879
		
24 h (n = 147)	(n = 47)	(n = 100)
	let-7b, miR-101a, miR-125a-3p, miR-135a*, miR-139-3p, miR-142-5p, miR-146b*, miR-181a, miR-186*, miR-193, miR-195, miR-203, miR-212, miR-297c, miR-30b*, miR-30c-2*, miR-335-5p, miR-362-5p, miR-380-3p, miR-382, miR-421, miR-448, miR-423-5p, miR-452, miR-466c-5p, miR-467b*, miR-483, miR-509-5p, miR-568, miR-542-3p, miR-574-3p, miR-574-5p, miR-669a, miR-670, miR-671-5p, miR-680, miR-689, miR-709, miR-712, miR-719, miR-720, miR-721, miR-759, miR-802, miR-876-3p, miR-882, miR-883a-5p	let-7a, let-7d, let-7e, let-7f, let-7g, let-7i, miR-101b, miR-103, miR-107, miR-10a, miR-10b, miR-122, miR-125a-5p, miR-125b-5p, miR-126-3p, miR-130a, miR-140*, miR-144, miR-148b, miR-151-5p, miR-155, miR-15a, miR-15b, miR-16, miR-17*, miR-183, miR-186, miR-18a, miR-190, miR-193b, miR-196b, miR-199a-3p, miR-199a-5p, miR-199b*, miR-19a, miR-208b, miR-20a, miR-21, miR-214, miR-22, miR-221, miR-222, miR-23a, miR-23b, miR-24, miR-25, miR-26a, miR-26b, miR-27a, miR-29a, miR-29b, miR-29b*, miR-29c, miR-301a, miR-30a, miR-30d, miR-30e, miR-31, miR-322, miR-323-3p, miR-324-5p, miR-331-3p, miR-335-3p, miR-338-3p, miR-338-5p, miR-340-5p, miR-342-3p, miR-345-5p, miR-34c, miR-350, miR-361, miR-365, miR-367, miR-369-3p, miR-370, miR-374, miR-376b, miR-376c*,miR-378, miR-450b-3p, miR-453, miR-463, miR-466b-5p, miR-466f-3p, miR-487b, miR-500, miR-582-5p, miR-652, miR-674*, miR-682, miR-687, miR-690, miR-705, miR-706, miR-741, miR-92a*, miR-875-5p, miR-93, miR-96, miR-99b

Numbers of miRNAs whose expression was altered by NP are shown in parentheses.

miRNAs whose expression was altered at both time points are underlined.

We performed quantitative RT-PCR (qRT-PCR) to confirm the expression levels of selected miRNAs. We chose miR-135a* and miR-199a-5p, two miRNAs that were found in microarray analyses to be highly up- and down-regulated, respectively. qRT-PCR results showed that miR-135a* was up-regulated 1.9-fold and miR-199a-5p down-regulated approximately 1.7-fold in independent experiments (Additional file 2, Figure S2). These up- or down expression patterns concurred with, but were more modest than, those detected in microarray analyses. The differences in the fold changes between microarray and qRT-PCR analyses may have been caused by differences in the principle between hybridization- and amplification-based methods. It can be also arisen from the inherently different gene sets during normalization between microarray and qRT-PCR. Several reports showed that the number of true calls is greater than those calculated using qRT-PCR if the correlation is low between microarray and qRT-PCR [24]. The miRNAs can appear to be better-expressed in microarray analysis because microarray experiments employ normalization techniques within the miRNA population not by one reference gene.

**Figure 3:**
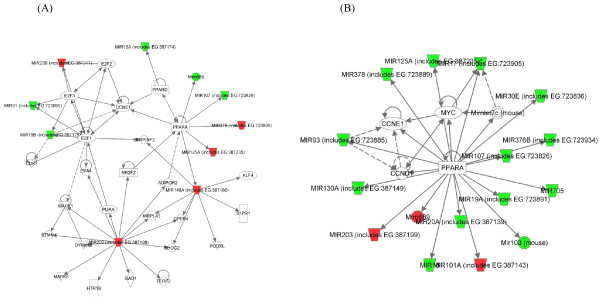
**Network analysis of miRNAs the expression of which in TM4 cells was altered by NP (A) 3 h. (B) 24 h**. Network analysis was performed using an algorithm supported by IPA. A best-matched network map was created.

Additionally, we created networks of differentially expressed miRNAs (Figure 3). At both 3 and 24 h post-NP treatment, *Ppara *was found to be a core miRNA-regulated gene. As mentioned above, Shah et al. reported that activated PPARA regulates miRNA expression in the mouse liver [23]. In TM4 cells exposed to NP, *Ppara *was down-regulated at both 3 and 24 h. We thus surmised that miRNAs regulated by *Ppara *may include miR-378, miR-125a-3p, and miRNA-148a at 3 h, and miR-20a, miR-203, and miR-101a at 24 h.

### Gene expression profiling of NP-treated TM4 cells

To evaluate the transcriptional regulation of target genes for miRNAs, we performed gene expression profiling using Agilent mouse whole genome 4 × 44 K arrays. The data generated were further combined with miRNA expression data in each time point. Each data set consisted of data from three samples each from NP-treated and control cells for each time point (3 and 24 h). At 3 and 24 h, 680 and 1,787 genes, respectively, were differentially expressed in NP-treated and control cells (fold-change > 2; *P *< 0.05, Welch's *t*-test). Of the genes the expression of which was altered in cells exposed to NP, 291 genes exhibited altered expression at both time points tested. We also analyzed gene expression using hierarchical clustering (evaluated by 1-way ANOVA; *P *< 0.05) to allow visual biological interpretation of the gene expression patterns. The results of this clustering analysis showed that the samples clustered according to time point and that gene expression patterns varied according to length of exposure to NP (Additional file 3, Figure S3). Functional analysis of the differentially expressed genes was performed using the GO database. These results are shown in Table 2. In total, 535 and 1,559 genes the expression of which was altered in cells treated with NP for 3 and 24 h, respectively, were functionally annotated with GO terms. The expression of genes with roles in signal transduction, transport, and transcription was highly altered in cells treated with NP for 3 h. The expression of genes with roles in growth, homeostasis, gametogenesis, spermatogenesis, and behavior was significantly altered in cells treated with NP for 24 h comparing to that of 3 h treated cells. Notably, the numbers of differentially expressed genes with roles in gametogenesis and spermatogenesis were highly increased at 24 h. Among genes with roles in gametogenesis and spermatogenesis, *Rps6ka2 *(ribosomal protein S6 kinase, polypeptide 2), *Afp *(alpha fetoprotein), *Tbpl1 *(TATAbox binding protein-like 1), and *Mast2 *(15 days embryo head cDNA) were up-regulated in NP-treated TM4 cells, while *Tssk1 *(testis-specific serine kinase 1), *Ereg *(epiregulin), and *Adam25 *(testase 2) were down-regulated in NP-treated TM4 cells.

**Table 2:** Functional classification of genes the expression of which in TM4 cells was modulated by NP

Function	No. of genes	
	3 h	24 h
Transport	77	278
Signal transduction	102	250
Transcription	73	169
Cell differentiation	38	119
Cell cycle	28	100
Response to stress	38	78
Lipid metabolism	20	75
Apoptosis	28	70
Cell adhesion	23	70
Behavior	11	59*
Cell proliferation	19	54
Protein biosynthesis	18	54
Immune response	20	42
Angiogenesis	10	29
Homeostasis	7	28
Growth	6	26
Cell-cell signaling	7	19
Gametogenesis	3	17*
Spermatogenesis	1	12*
Inflammatory response	6	10
**Total number of genes**	**535**	**1,559**

*Number of genes hit into the categories remarkably increased (over 5-fold) at 24 h comparing to at 3 h

### Comprehensive prediction of target genes for deregulated miRNAs

To comprehensively identify target genes, we used the GeneSpring GX v11.0 software. It is known that miRNAs target multiple genes and that target genes can be regulated by negative or positive feedback mechanisms. Before analyzing the target genes for miRNAs the expression of which was altered in NP-treated cells, we filtered the target gene set to include only genes the expression of which showed the opposite pattern to that of the corresponding miRNAs. In total, 338 and 1,024 miRNA target genes were down-regulated at 3 and 24 h post-NP treatment, respectively (with the corresponding miRNAs being up-regulated). 146 and 3,128 target genes were up-regulated at 3 and 24 h post-NP treatment (with the corresponding miRNAs being down-regulated).

**Figure 4:**
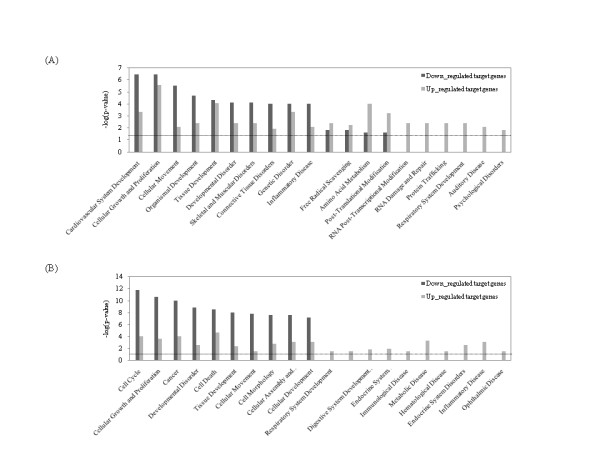
**The top 10 ranked biological functions miRNA target genes**. **(A) **Target genes the expression of which was down- or up-regulated at 3 h **(B) **Target genes the expression of which was down- or up-regulated at 24 h. The dotted line indicates the threshold for statistical significance (*P *< 0.05).

The detected target genes were categorized according to biological function using Ingenuity Pathway Analysis (IPA) software. The top-ranked biological functions of miRNA target genes in NP-treated TM4 cells are shown in Figure 4. Biofunctional analysis showed that target genes for differentially regulated miRNAs were, as expected, primarily involved in the control of cellular growth and proliferation, the cell cycle, and cell death. At 3 h post-NP-treatment, target genes implicated in growth and proliferation, tissue development, and genetic disorders were down-regulated by miRNAs, while several genes linked to RNA damage and repair were up-regulated to repair the cellular damage sustained. Because of the cytotoxic effects of NP, most target genes related to cell cycle or cell death were differentially expressed at 24 h post-NP treatment.

### Functional analysis of miR-135a* target genes

miR-135a* was significantly up-regulated after exposure to NP for 24 h. The change in miR-135a* expression was the highest in this study (20.6-fold up-regulation). We thus suggest that miR-135a* may play important roles following NP exposure. Through categorization of its target genes, we identified its potential molecular and cellular roles as being cell cycle, cell death, cell morphology, cell-to-cell signaling and interaction, and cellular assembly and organization (Table 3). Down-regulated miR-135a* target genes are listed in Table 4. Canonical pathway analysis of these genes showed that the expression of genes linked to the production of nitric oxide (NO) and reactive oxygen species (ROS; *Ncf2 *and *Ppp2r2b*), Wnt/β-catenin signaling (*Wnt1*), and ERK/MAP kinase signaling (*Rapget3*) could be down-regulated by miR-135a* in response to NP exposure

**Table 3:** Functional classification of miR-135a* target genes

Molecular and cellular functions		
**Name**	***P*-value**	**No. of genes**

Cell Cycle	2.48E-04 - 1.72E-02	7
Cell Death	1.02E-03 - 4.78E-02	7
Cell Morphology	1.02E-03 - 4.69E-02	3
Cell-To-Cell Signaling and Interaction	1.02E-03 - 4.78E-02	4
Cellular Assembly and Organization	1.02E-03 - 4.00E-02	2

**Table 4:** miR-135a* target genes the expression of which was down-regulated in NP-treated TM4 cells

Gene symbol	Gene title	Acc. No	Fold change	GO category
*Nasp*	Nuclear autoantigenic sperm protein (histone-binding)	NM_016777	-1.05	Cell cycle, Cell proliferation
*Rapgef3*	Rap guanine nucleotide exchange factor (GEF) 3	NM_144850	-1.82	Intracellular signaling cascade
*Ncf2*	Neutrophil cytosolic factor 2	NM_010877	-1.43	Superoxide metabolic process
*Ywhaq*	Tyrosine 3-monooxygenase/tryptophan 5-monooxygenase activation protein, theta polypeptide	AK170335	-1.82	Signal transduction
*E330013P04Rik*	RIKEN cDNA E330013P04 gene	BC076607	-1.08	
*Ddah1*	Dimethylarginine dimethylaminohydrolase 1	NM_026993	-1.55	Nitric oxide biosynthetic process
*Ppp2r2b*	Protein phosphatase 2, regulatory subunit B, beta isoform	NM_028392	-1.69	Signal transduction
*Cyp2c40*	Cytochrome P450, family 2, subfamily c, polypeptide 40	NM_010004	-1.46	Oxidation reduction
*Rttn*	Rotatin	AK015013	-1.11	Multicellular organismal development
*Slc10a7*	Solute carrier family 10, member 7	NM_029736	-1.22	Ion transport
*Aspm*	Asp (abnormal spindle)-like, microcephaly associated	NM_009791	-1.82	Cell cycle
*Tmem108*	Transmembrane protein 108	AK039631	-1.98	Biological process unknown
*Tpm1*	Tropomyosin 1, alpha	NM_024427	-1.84	Embryonic development
*1700074P13Rik*	RIKEN cDNA 1700074P13 gene	NM_028550	-1.55	Biological process unknown
*Wnt1*	Wingless-related MMTV integration site 1	NM_021279	-1.17	Wnt receptor signaling pathway
*Crip3*	Cysteine-rich protein 3	NM_053250	-1.33	T cell proliferation
*Zfr*	Zinc finger RNA binding protein	AK037578	-1.06	Multicellular organismal development
*Mmp1a*	Matrix metallopeptidase 1a (interstitial collagenase)	NM_032006	-1.04	Proteolysis, Collagen catabolic process
*Olfr1309*	Olfactory receptor 1309	NM_146447	-1.49	Signal transduction
*Nfic*	Nuclear factor I/C	NM_008688	-1.17	Regulation of transcription

In the canonical pathway, its candidate targets fall into several categories. Genes implicated in the production of NO and ROS were predicted to be miR-135a* targets. There have been reports that NP suppresses cell growth and cellular respiration in yeast cells by inducing ROS generation, and the formation of hydroxyl radicals in rat brain striatum [25,26]. Recently, NP was shown to induce ROS generation in human blood neutrophils and rat Sertoli cells through the activation of signal transduction pathways [20,27]. NP increases ROS levels and lipid peroxidation and decreases the activity of antioxidant enzymes in the rat testis [28]. The results of the present study further suggest that mouse Sertoli cells are also affected by NP-induced ROS. We do not know the precise mechanism of NP-induced ROS generation in mouse Sertoli cells; this subject requires further investigation.

Other predicted target genes influence Wnt/β-catenin signaling. The Wnt/β-catenin signaling pathway is known to play fundamental roles in the regulation of proliferation, regeneration, and differentiation in many tissues and types of cells, including stem cells. Although there have been few reports on possible relationships between NP exposure and Wnt/β-catenin signaling, we suggest that key cell functions may be affected by altered expression of Wnt/β-catenin signaling pathway-related genes.

miR-135a* may target ERK/MAP kinase signaling-related genes. MAP kinases are known to be key regulators of cell differentiation, proliferation and apoptosis [29]. A study showed that 17β-estradiol and structurally diverse estrogenic compounds, including bisphenol A and NP, activated MAP kinases in MCF-7 cells [30]. Further, the three major MAPK subfamilies (ERK, JNK, and p38 MAPK) were activated by NP in SCM1 human gastric cancer cells. Notably, NP induced apoptosis by activating a Ca^2+^- and p38 MAPK-dependent signaling pathway. Although the effects of ERK during cell death remain unclear, there is a suggestion that it may promote cell survival [31]. Indeed, the activation of ERK was shown to increase neuronal survival in retinal ganglion cells [32]. Based on the results of these previous studies, we suggest that the activation of ERK/MAP kinase signaling may also promote the survival of Sertoli cells. We believe that these tentative conclusions warrant further investigation and hope that future studies we will increase our understanding of the mechanisms of NP-induced miRNA-mediated cytotoxicity in mouse Sertoli cells.

## Conclusions

Recently, it has been reported that xenoestrogenic compounds, including NP, cause reproductive toxicity in males (e.g., a decline in semen quality). Here, to evaluate the mode of action of NP on the male reproductive system, we comprehensively evaluated mRNA and miRNAs expression profiles in NP-exposed mouse TM4 Sertoli cells through microarray analysis. Exposure of TM4 Sertoli cells to NP significantly altered miRNA expression levels. By analyzing the biofunctions of both deregulated miRNAs and their target genes, we obtained information about genes regulated by miRNAs. Additionally, core miRNAs and target genes deregulated in NP-treated cells were identified through functional and network analyses of miRNAs and target genes. Although further studies are required to determine the function of these selected miRNAs, this information may aid in understanding the epigenomic regulation of toxicity in Sertoli cells exposed to NP and could support the risk assessment of environmental estrogenic compounds.

## Competing interests

The authors declare that they have no competing interests.

## Authors' contributions

JC analyzed the microarray data and drafted the manuscript. JO designed and performed the overall experiments and helped to finalize the manuscript. MC, HP, SP and SK contributed to the sampling and performed the microarray experiments. SK performed the real-time PCR for validation and participated in statistical analysis. MO and SH participated in overall design of microarray analysis. SY designed and coordinated the overall study as a corresponding author and helped to draft the manuscript. All authors read and approved the final manuscript.

## Supplementary Material

Additional file 1 **Supplemental Figure S1: Hierarchical clustering of miRNA expression profiles in NP-treated TM4 cells**. Click here for file

Additional file 2 Supplemental Figure S2: Validation of miR-135* and miR-199a-5a levels by qRT-PCR. Click here for file

Additional file 3 **Supplemental Figure S3: Hierarchical clustering of the gene expression profiles in NP-treated TM4 cells**. Click here for file
