# Supplementary material for: miRNA regulation of cytotoxic effects in mouse Sertoli cells exposed to nonylphenol
Source: Reprod Biol Endocrinol. 2011 Sep 14;9:126. doi: 10.1186/1477-7827-9-126 (PMC3196907; doi:10.1186/1477-7827-9-126)
Supplement: Additional file 1 — Supplemental Figure S1: Hierarchical clustering of miRNA expression profiles in NP-treated TM4 cells. [file 1477-7827-9-126-S1.PDF]

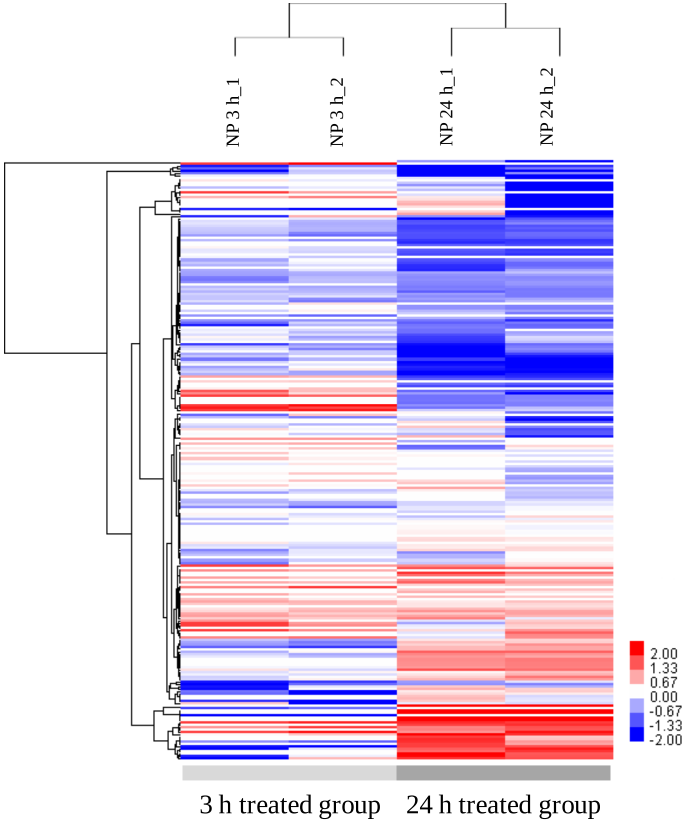

Supplemental Figure S1. Hierarchical clustering of miRNA expression profiles in NP-treated TM4 cells
