# Supplementary material for: miRNA regulation of cytotoxic effects in mouse Sertoli cells exposed to nonylphenol
Source: Reprod Biol Endocrinol. 2011 Sep 14;9:126. doi: 10.1186/1477-7827-9-126 (PMC3196907; doi:10.1186/1477-7827-9-126)
Supplement: Additional file 2 — Supplemental Figure S2: Validation of miR-135* and miR-199a-5a levels by qRT-PCR. [file 1477-7827-9-126-S2.PDF]

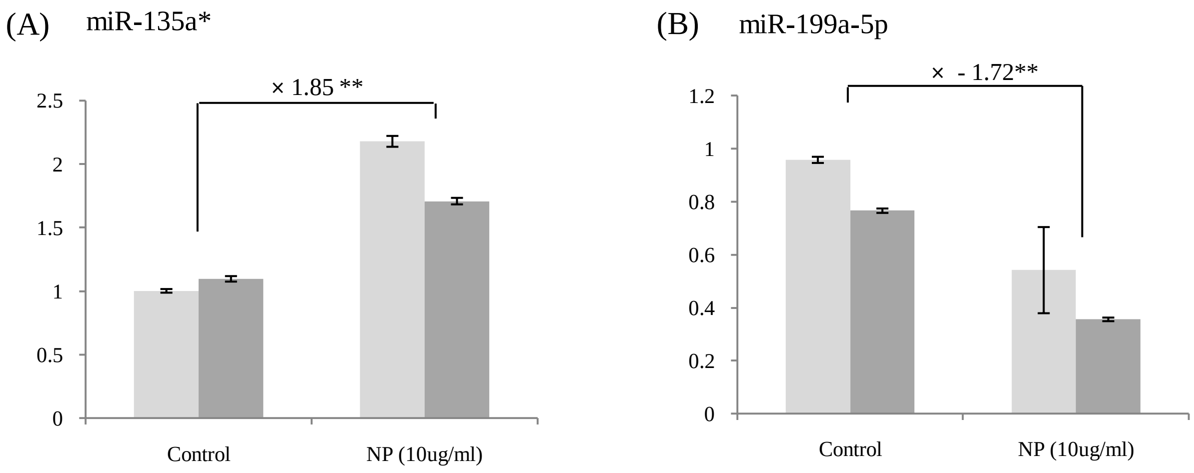

Supplemental Figure S2. Validation of miR-135* and miR-199a-5a levels by qRT-PCR. Data are presented as the mean ± SEM of biological triplicates. **$P < 0.01$, vs. control.
