# Supplementary material for: miRNA regulation of cytotoxic effects in mouse Sertoli cells exposed to nonylphenol
Source: Reprod Biol Endocrinol. 2011 Sep 14;9:126. doi: 10.1186/1477-7827-9-126 (PMC3196907; doi:10.1186/1477-7827-9-126)
Supplement: Additional file 3 — Supplemental Figure S3: Hierarchical clustering of the gene expression profiles in NP-treated TM4 cells. [file 1477-7827-9-126-S3.PDF]

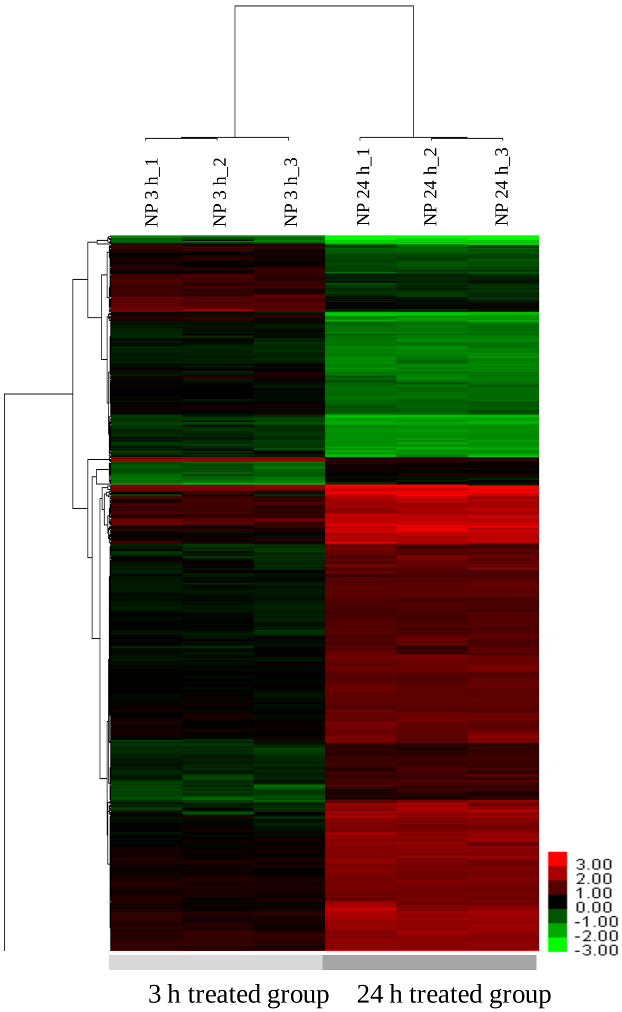


**Supplemental Figure S3.** Hierarchical clustering of the gene expression profiles in NP-treated TM4 cells
